# What is sexual wellbeing and why does it matter for public health?

**DOI:** 10.1016/S2468-2667(21)00099-2

**Published:** 2021-06-22

**Authors:** Kirstin R Mitchell, Ruth Lewis, Lucia F O’Sullivan, J Dennis Fortenberry

**Affiliations:** MRC/CSO Social and Public Health Sciences Unit, University of Glasgow, Glasgow, UK; Psychology Department, University of New Brunswick, NB, Canada; Indiana School of Medicine, Indiana University, IN, USA

## Abstract

Sexual health has provided a guiding framework for addressing sexuality in public health for several decades. Although the WHO definition of sexual health is revolutionary in acknowledging positive sexuality, public health approaches remain focused on risk and adverse outcomes. The long-standing conflation of sexual health and sexual wellbeing has affected our ability to address everyday sexual issues. This Viewpoint provides a way forward to resolve this impasse. We propose sexual wellbeing as a distinct and revolutionary concept that can be operationalised as a seven-domain model. We situate sexual wellbeing alongside sexual health, sexual justice, and sexual pleasure as one of four pillars of public health enquiry. We argue that sexual wellbeing is imperative to public health as a marker of health equity, a meaningful population indicator of wellbeing, a means to capture population trends distinct from sexual health, and an opportunity to refocus the ethics, form, and practices of public health.

## Introduction

The widely-adopted definition of sexual health developed by WHO is expansive. It includes concepts such as the absence of disease and coercion, and draws attention to sexual rights and to the possibility of sexual pleasure.^1^ Yet, wellbeing is mentioned as an adjunctive element of sexual health; the unique elements of wellbeing— distinct from sexual health—are not identified.

The WHO definition of sexual health reduces stigma by helping researchers, educators, clinicians, and policy makers acknowledge positive sexuality and sexual experiences as key public health outcomes. However, public health approaches to sexuality remain rooted firmly in the medical and biological sectors, with their focus largely on adverse health outcomes and concomitant risks. This risk-focused approach has come to be viewed as the standard for public health, eclipsing other aspects of sexuality, even though health is seldom—if ever—the primary reason for engaging sex.^2^ Such a public health vision overlooks a contemporary body of evidence from scientific research supporting perspectives far broader than those associated with sexual health.^3^ In practice, perspectives on what constitutes normal sexuality are understood through a public health lens.^4^ In research, confusion and inconsistency across different studies, often in reference to the same issues, make it difficult to advance the science in this area. Most importantly, the conflation of sexual wellbeing and sexual health obscures the diversity of experiences—not clearly addressed in definitions of sexual health—that people identify as relevant to their broader wellbeing. This truncated perspective ultimately limits our ability to understand and address everyday sexual issues.

The absence of a sharp distinction between sexual wellbeing and sexual health has created ambiguity in policy rhetoric, and hindered conceptualisation of sexual wellbeing as a valid outcome of public health interventions. For more than a decade, advocates and thought leaders have acknowledged the need to expand the scope of inquiry and intervention in public health from a singular focus on sexual health to attention on sexual wellbeing as a distinct concept. The call to make this shift partly emerged from a WHO–UN Population Fund meeting in 2007. At that time there was considerable difficulty agreeing on the meaning of sexual wellbeing. Since then, efforts to adopt sexual wellbeing as part of a comprehensive, holistic, and progressive goal for public health have stalled indefinitely, awaiting additional justification, articulation, and operationalisation.

This Viewpoint constitutes a long overdue effort to effectively resolve this impasse. We build on increasing awareness of the limits and constraints of a sole focus on sexual health, and an emerging body of research on the relevance of sexuality to wellbeing. This emerging interest mirrors the greater attention given to population wellbeing of late. A more nuanced, multi-dimensional framework is required.

Our conceptualisation of sexual wellbeing resonates with the biopsychosocial–cultural framework based in people’s perspectives on sexual wellbeing in mid and later life.^5^ This perspective locates sexual wellbeing firmly in relation to sexual health, and in relation to two other pillars of public health focused inquiry—sexual pleasure and sexual justice—each needed to address structural determinants of sexual inequities (figure). Here, we describe the interconnections of these four pillars and the conceptual overlap with sexual wellbeing.

A guiding premise of our argument is that the concept of sexual wellbeing demands recognition, not as an extension, subclass, or alternate form of sexual health, but as a distinct and revolutionary concept that challenges our accepted thinking, and has far-reaching global applications in public health that have been neglected to date. However, our interest in expanding recognition of the public health relevance of sexual wellbeing does not undermine the importance of sexual health, sexual pleasure, and sexual rights. Rather, we believe sexual wellbeing brings conceptual clarity to our shared understanding of sexual health, identifies areas of conceptual difference, and clarifies a much broader public health perspective on sexuality beyond sexual health alone.

## Four pillars for a comprehensive public health approach to sexuality

### Sexual health

Our model follows key issues identified in the WHO definition of sexual health: fertility regulation, prevention and management of sexually transmitted infections (STIs; including HIV), sexual violence prevention, and sexual functions (including sexual desire and arousal).^6^ The relevance of these issues to global public health were underlined by the 2018 Guttmacher–*Lancet* Commission on sexual and reproductive health and rights focusing on the role of the Sustainable Development Goals in promoting specific areas of sexual health.^7^ The WHO Working Group to Operationalize Sexual Health^8^ explicitly linked these aspects of sexual health to “physical, emotional, mental, and social wellbeing in relation to sexuality”, centred within an interconnected framework of sexual health influences, including attention to human rights and positive approaches to sexuality.

### Sexual pleasure

Sexual pleasure is related to both sexual health and sexual wellbeing but its distinct relevance to public health is increasingly recognised.^9,10^ A recent definition of sexual pleasure addresses the diverse physical and psychological satisfactions of sexual experience, and key enabling factors, such as self-determination, consent, safety, privacy, confidence, and the ability to communicate and negotiate sexual relations.^11^ Furthermore, this definition specifies that pleasure requires fundamental social and cultural conditions of sexual rights in terms of equality, non-discrimination, autonomy, bodily integrity, and freedom of expression. To improve the operationalisation of sexual pleasure, we propose the inclusion of two key elements (figure): events (eg, key features of a sexual occasion, such as the repertoire, timing, and spacing of different sexual practices, occurrence of orgasm, use of a condom or contraception) and people (eg, interactional elements of sexual pleasure, encompassing interpersonal dynamics, such as communication, negotiation, and trust). These elements illustrate the conceptual relationships of pleasure with sexual health and sexual wellbeing, and help to summarise the diverse factors associated with sexual pleasure without privileging pleasure as the cornerstone of wellbeing.^12,13^

### Sexual justice

Sexual justice represents larger global efforts to ensure social, cultural, and legal supports for equitable, person-centred sexual and reproductive experiences. Public health plays an instrumental role in documentation and mitigation of adverse outcomes associated with disparities in human rights. Public health also contributes to the promotion of equal access to distributive and restorative justice, helping combat historical restrictions of sexual citizenship on the basis of ethnicity, sex, and sexual and gender identity.^7,14^ Among many specific examples, public health has played a central role in addressing violence and discrimination linked to sexuality among people living with HIV.^15^ With regard to the pillar of sexual justice, we propose trauma-informed, sex positive public health practices as a specific tool for enacting social justice. This practice implies restorative approaches that acknowledge and address adverse sexual experiences, trauma that resonates through the life course, and the effects on sexual wellbeing.^16,17^ Sex positivity is central to a public health relevant concept of sexual wellbeing. Trauma-informed, sex positive practices refer to perspectives and approaches that emphasise contributions of sexuality and sexual expression to overall wellbeing.

### Sexual wellbeing in the context of sexual health, sexual pleasure, and sexual justice

We believe it is now imperative for the field of public health to adopt and integrate sexual wellbeing in efforts to address pervasive inequities related to sexuality and sexual behaviour, in particular, those driven by gender and sexual identity. Our framework allows distinct attention to the role of sexual wellbeing in overall wellbeing^18^ and better support for operationalisation, measurement, and potential public health intervention.

Operationalisation and measurement of sexual wellbeing is challenged by diverse perspectives on its definition and meaning. Outside of professional spheres, people rarely refer to sexual wellbeing per se, although the concept is inferred in the idea of a sex life that is supposedly good or going well. Pharmacies sometimes sell products, such as vaginal tightening gel, nutritional supplements, and fertility aids, under the banner of sexual wellbeing. This commodification probably influences public understanding and focuses attention on a narrow set of assessment criteria to judge whether a sex life is going well.^19^ However, definitions of sexual wellbeing in academic literature attend to a broader range of aspects. Several measures have been developed, which inlclude unidimensional measures defined in terms of a global assessment of one’s sex life.^20^ Laumann and colleagues defined sexual wellbeing as “the cognitive and emotional evaluation of an individual’s sexuality”, and used four satisfaction judgments.^21^ Muise and colleagues used a similar definition, but extended the domains to include satisfaction with sexual relationships and functioning, sexual awareness, sexual self-esteem, and body image esteem.^22^ Syme and colleagues, in a study of sexuality in mid and later life, referred to sexual wellness with four dimensions: psychological (eg, cognitions, emotions, and concepts), social (eg, relationship and shared experience), biological and behavioural (eg, functioning, behaviours and scripted sexual activities), and cultural (eg, age or time in life, and gender and sexual orientation).^5^

In operationalising sexual wellbeing, we developed a model with seven core domains: sexual safety and security, sexual respect, sexual self-esteem, resilience in relation to past sexual experiences, forgiveness of past sexual events, self-determination in one’s sex life, and comfort with one’s sexuality (table). Domains were identified and refined through intensive engagement with wide-ranging literature, including a review of sexual wellbeing definitions and measures.^62^ In developing this new concept of sexual wellbeing, we specified five criteria: (1) the concept should be distinct from sexual health, sexual satisfaction, sexual pleasure, and sexual function; (2) the concept should be applicable to people regardless of whether they are sexually active; (3) the concept should apply to people irrespective of their partnership status (including those who are unpartnered); (4) the concept should be based in elements amenable to change through policy, public health action, clinical support, or personal growth; and (5) the concept should focus both on a person’s summation of experience and assessment of prospects for sexual wellbeing in the near future. For each of the seven domains, we provide a working definition and show its contribution to sexual wellbeing, provide examples of relevance to public health, and offer examples of how the domain might be operationalised (table).

## Why is sexual wellbeing imperative to public health?

We anticipate some resistance to considering wellbeing as a valid goal of public health. Critics refer to the subjective and variable qualities of wellbeing, necessarily influenced by social and cultural contexts, and played out in individual attitudes and actions.^63^ Introduction of surveillance infrastructures and goals in diverse national and cultural settings will require persistence and careful data gathering. For example, the COVID-19 pandemic and the life-changing interruptions of migration have changed sexual priorities such that even delimiting appropriate sexual wellbeing timeframes is challenging.^64,65^ There might be resource constraints to monitoring that requires a multi-dimensional measure, and political resistance to giving prominence to sexual wellbeing alongside risk-focused outcomes. Acknowledging these issues, we set out four ways in which sexual wellbeing is highly relevant to core functions of contemporary public health.

### Sexual wellbeing is a marker of health equity

Within the field of public health, population wellbeing approaches seek to establish measurable and achievable goals toward equity.^66^ Sexual wellbeing is an appropriate marker of population wellbeing, given inequities related to sexuality and sexual expression. These inequities include systemic and pervasive racial, ethnic, or immigrationbased discrimination, gender-based violence, sexual identity-based violence, and STIs and HIV.^7^ A sexual wellbeing approach recognises the transgenerational traumas that mark the unique needs of marginalised people. This recognition then supports implementation of population health approaches that are anti-oppressive, intersectional, and culturally and contextually adapted.^67,68^

### Sexual wellbeing is a meaningful population indicator of wellbeing

As population wellbeing continues to be an aspirational goal of public health, sexual wellbeing emerges as an important component of overall wellbeing. A populationbased study has shown the positive contribution of a measure of sexual wellbeing in population surveys.^18^ Sexual wellbeing provides important insights into population wellbeing over the entire life course. Data on sexual wellbeing would add new dimensions to community engagement in health issues, address structural determinants of health at local levels, and link local and larger public health policy and practice related to sexual and reproductive health.^69^

### Sexual wellbeing captures population trends distinct from sexual health measures

Sexual wellbeing incorporates outcomes that are increasingly recognised as important to, but distinct from, biomedically focused sexual health intervention. In a review of English-language sexual health promotion interventions published from 2010–14, four of 33 interventions’ stated goals were related to sexual wellbeing; most interventions (n=28) either targeted sexual wellbeing in addition to biomedical sexual health, or addressed sexual health outcomes through a focus on sexual wellbeing.^70^ Tracking sexual wellbeing also shows key population trends in the importance of sex to broader wellbeing. For example, in four decades of French national surveys (1970s to 2006), the proportion of people reporting that sexual intercourse was considered essential to feeling good about oneself increased from 48% to 60% for women and from 55% to 69% for men.^71^

### Sexual wellbeing refocuses the ethics, form, and practices of public health

Positioning sexual wellbeing as a driver for cross-cutting public health innovation challenges the structural origins of sexual inequities and requires acknowledging that sexual wellbeing is experienced by people in relation to contexts and surroundings.^72^ This suggests that surveillance of sexual wellbeing, at individual and community levels, is required, and thus challenges the centrality of privacy in sexuality. Public health surveillance is well established in sexual health prevention and control (eg, for STIs). However, extension to sexual wellbeing refocuses such surveillance—and the capacity to offer intervention—into areas traditionally outside of public health function. We contend that such surveillance is necessary to focus resources on populations of greatest need, and to track those who enter or exit intervals of greater need.^73^ Such functions might require redefinition of relationships between communities and public health entities to create trust and full engagement while protecting privacy.^74^

## Conclusion

We believe that the adoption and integration of sexual wellbeing as an essential concept in efforts to address sexual inequities is imperative for the field of public health. A broad and consistent body of research supports the relevance of sexual wellbeing as a distinct correlate of sexual health whose importance has been obscured by conflation of the two concepts.^75^ Our conceptualisation of sexual wellbeing relates to sexual health and pleasure (a primary motivation for sex), and to social, cultural, and political frameworks of sexual justice. By identifying trauma-informed sex positivity as a central guiding public health value, we anchor our approach to sexual wellbeing with a corresponding recognition of the notable significance of both sexuality and sexual trauma in our lives. Inclusion of sexual wellbeing as a public health goal is attainable but requires an additional data-driven vision and specified objectives. Our initial steps toward an approach, and ultimately conceptualisation, of sexual wellbeing builds on existing measures and intentionally focuses on dimensions and an organisational structure that might be addressed effectively by public health policy and intervention.

The personal losses of illness and death, the threats to the health of families, the interpersonal effects of quarantine and physical distancing, and the pervasive economic consequences of the COVID-19 pandemic will almost certainly have enduring effects on sexual wellbeing that have yet to be fully described and appreciated. Significant public health resources will be needed, simply to meet the basic needs of many people. Our proposed shift in thinking about sexuality in public health will facilitate movement forward in the process of reorganising social structures to meet these effects, including attention to population wellbeing.

## Figures and Tables

**Figure:**
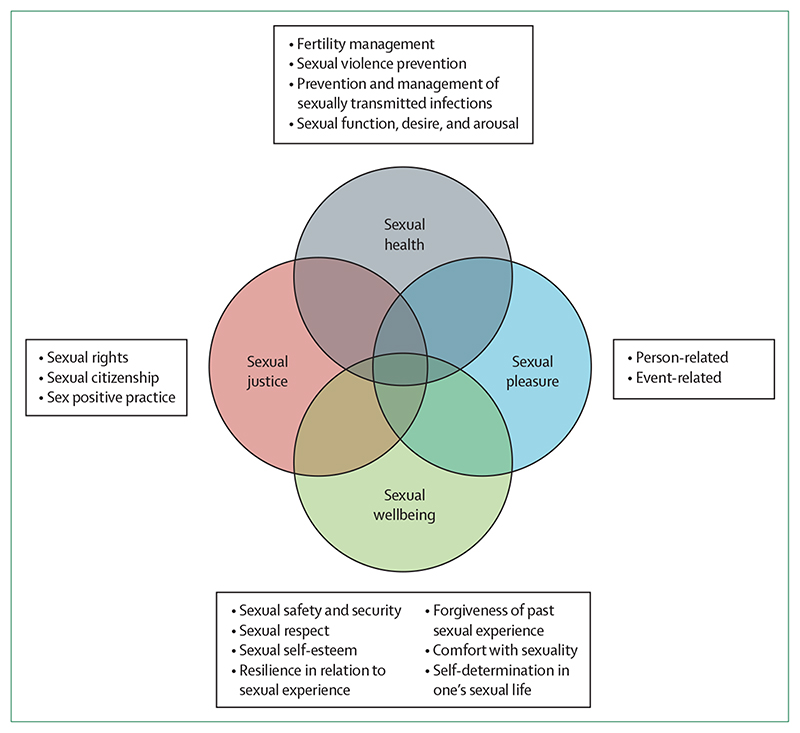
Four pillars of comprehensive public health focused inquiry and intervention in relation to sexuality

**Table:** Sexual domains of sexual wellbeing: definitions, contributions, relevance to public health, and potential operationalised measures

	Definition	Contribution to sexual wellbeing	Relevance to public health (examples)	Potential operationalised measures
Sexual safety and security^5,23^	Experience of reduced threat coupled with experience of actions taken to assuage vulnerability^24^	Free expression of sexuality;^25,26^ rituals of safety;^27^ relationship trust;^28^ coercion-free environments^29^	Gender-based violence;^30^ technologies for partner identification;^31^ sexual coercion;^28,32^ legal protections for sexual rights;^33,34^ teaching sexual consent; addressing risks to sex workers	Little worry about future sex life; absence of unwanted vulnerability during sexual activities; feeling safe with a sexual partner
Sexual respect^5^	Perception of positive regard by others for one’s sexual personhood^35^	Mitigates influence of experiences of violence;^36^ tolerance of differences;^37^ validation by others^38,39^	Elements of interventions to reduce sexual harassment (eg, in higher education^39^); sexual rights of people with minority identities and or marginalised experiences (eg, sexual minority groups living with HIV^40^)	Sexual identity and preferences accepted by those around you; sexual identity and preferences accepted by broader culture
Sexual selfesteem^5,22^	Affective appraisals of oneself as a sexual being^41^	Associated with sexual satisfaction;^42^ mindful attention to sexual interactions^43^	Interventions to improve overall sexual functioning;^44^ building capacities to sexually relate to a partner^22^	Feeling good about your body sexually; feeling in control of sexual thoughts and desires
Resilience in relation to sexual experiences	Maintenance of equilibrium in response to sexual stress, dysfunctions-adversity- or trauma^45^	Influences long-term trajectories of wellbeing;^46^ interplay of person’s resources, needs, and assets^3^	Lessens the effects of sexual minority stressors;^47^ support for recovery from emotional trauma	Having someone to talk to openly about your sex life; taking a long time to recover if something bad happens in your sex life
Forgiveness of past sexual experiences	Halted patterns of self-blame, self-stigmatisation, shame, avoidance, aggression, regret, and revenge^48,49^	Reduces harm and improves wellbeing;^50^ improves relationship quality;^51^ mitigates trauma of laws, policies, and practices that harm or do not prevent harm^52^	Interventions to support recovery from sexual trauma and improve subsequent health _outcomes_^50,53^	Forgiveness of yourself about mistakes made in past sex life; forgiveness of others about things they have done to you in past sex life
Self-determination in one’s sex life^5^	Free choice or rejection of sexual partner(s), behaviours, context and timing without pressure, force, or felt obligation^54^	Directly influences sexual wellbeing;^55^ autonomous choice about sexuality supports ability to orient choices toward others^56^	Global public health significance of unwanted sexual interactions;^7^ reproductive self-determination for women	Only doing sexual activities that you really want to do; not feeling pressure from others to do specific sexual activities
Comfort with sexuality^5^	Experience of ease in contemplation, communication, and enactments of sexuality and sex^57^	Exploration of sexual identities and experiences;^58^ associated with partner communication, trust, and forgiveness;^59^ mindfulness in attending to sexual contexts^43,60^	Comfort in sexual communication associated with improved sexual health behaviours such as contraceptive use;^61^ ease in discussing sexual anatomy with a health professional; alleviating sexual guilt	Feeling focused and experiencing a sense of flow during sexual activities; absence of unwanted thoughts during sexual activities; absence of shame about sexual thoughts and desires; feeling comfortable with your sexual identity and preferences; a pleasurable sex life

## Search strategy and selection criteria

Our initial search terms focused on “sexual wellbeing” using multiple databases, including Google Scholar, Psychinfo, and Ovid. No specific inclusion criteria were used other than relevance to emerging concepts. On the basis of this extensive process, we produced an initial set of sexual wellbeing domains summarised in the table. Additional literature reviews were based on key words “sexual safety,” “sexual security,” “sexual respect,” “sexual self-esteem,” “sexual resilience,” “sexual forgiveness,” “sexual self-determination,” and “sexual comfort.” No date limits were used in these reviews. Abstracts of retrieved articles were reviewed for relevance, with detailed review of selected papers, books, and book chapters. Additional resources were identified by hand searching the citation lists of relevant sources.
